# Adenocarcinoma with mediastinal lymph node involvement developed from a pure ground grass nodule during 14 years

**DOI:** 10.1002/rcr2.1152

**Published:** 2023-04-25

**Authors:** Hiroyuki Miura, Jun Miura, Shinichi Goto, Tomoko Yamamoto

**Affiliations:** ^1^ Department of Thoracic Surgery Akiru Municipal Medical Centre Tokyo Japan; ^2^ Department of Surgery Kyorin University School of Medicine Tokyo Japan; ^3^ Department of Respirology Akiru Municipal Medical Centre Tokyo Japan; ^4^ Department of Pathology Tokyo Women's Medical University Tokyo Japan

**Keywords:** computed tomography, long‐term follow‐up, lung adenocarcinoma, pure ground grass nodule, tumour progression

## Abstract

A 69‐year‐old female Japanese patient presented with an abnormal shadow on chest computed tomography (CT). She had received a mastectomy 14 years prior. Under the diagnosis of primary lung cancer, left upper lobectomy was conducted. Pathology showed a lepidic adenocarcinoma with mediastinal lymph node metastases with pT2aN2M0. Upon retrospective analysis, the chest CT at the time of mastectomy depicted a ground‐glass nodule (GGN) of less than 20 mm. Over the previous 10.5 years, the concentration of the central part of the GGN increased. Conclusively, a pure GGN developed into lung adenocarcinoma with mediastinal lymph node involvement over 14 years. She had bone metastases 4 years after the lobectomy but has survived for five and a half years after surgery with treatment with osimertinib. Comparison readings of films should be performed throughout the patient's clinical history to detect subtle shadow alterations indicative of tumour progression.

## Graphical abstract

We had an interesting case of a pure ground‐glass nodule that developed into lung adenocarcinoma with mediastinal lymph node involvement over 14 years. Here we report the temporal change in the opacity of the GGN and the concept of image interpretation.

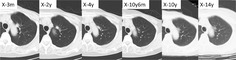

## INTRODUCTION

A prevailing theory states that adenocarcinoma in situ proceeds stepwise into invasive adenocarcinoma, there are a few cases that have emerged from long‐term observation of a pure ground‐glass nodule (GGN). We had an interesting case that developed into lung adenocarcinoma with mediastinal lymph node involvement over 14 years. Here we report the temporal change in the opacity of the GGN and the concept of image interpretation.

## CASE REPORT

**FIGURE 1:**
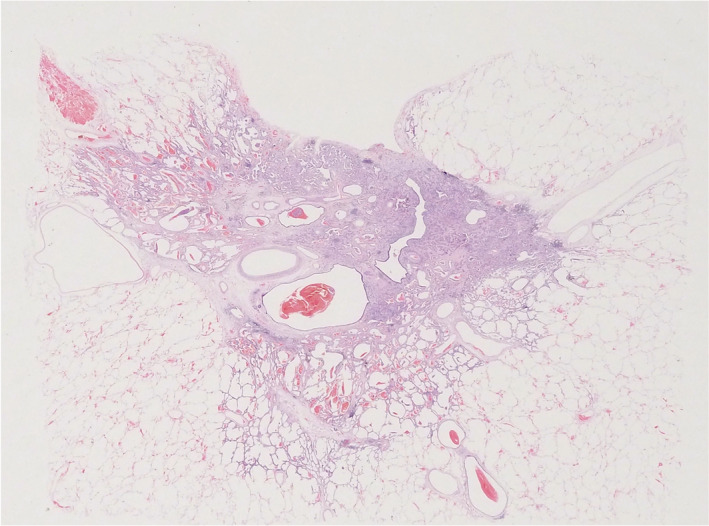
Loupe image of the lung tumour indicating lepidic adenocarcinoma. (HE stain)

A 69‐year‐old woman admitted with an abnormal shadow on chest computed tomography (CT). The patient was a non‐smoker. She received a left mastectomy for breast cancer 14 years previously. She underwent an annual check‐up CT to screen for breast cancer metastases. The chest CT revealed a mixed GGN (4.4 × 2.2 × 1.6 cm in size) in the left S3c area. Positron emission tomography revealed accumulation (tumour) on the left lung with a maximum standardized uptake value of 2.3. Blood work showed elevated carcinoembryonic antigen (9.3 ng/mL) and sialyl Lewis‐x antigen (46 U/mL). The hemogram and renal and hepatic functions were within the normal ranges. Under the diagnosis of primary lung cancer, a left upper lobectomy was conducted. Pathology showed a 3.7 × 2.6 × 2.3 cm (invasive dimension of 2.1 × 1.7 cm) tumour with visceral pleural invasion, which was a lepidic adenocarcinoma (Figure 1) with lymph node metastases comprising subaortic and paraaortic lymph nodes. The pathological stage was IIIA with pT2aN2M0. Molecular analysis revealed exon 19 deletion. Four courses of adjuvant chemotherapy with cisplatin and vinorelbine detartrate were performed.

**FIGURE 2:**
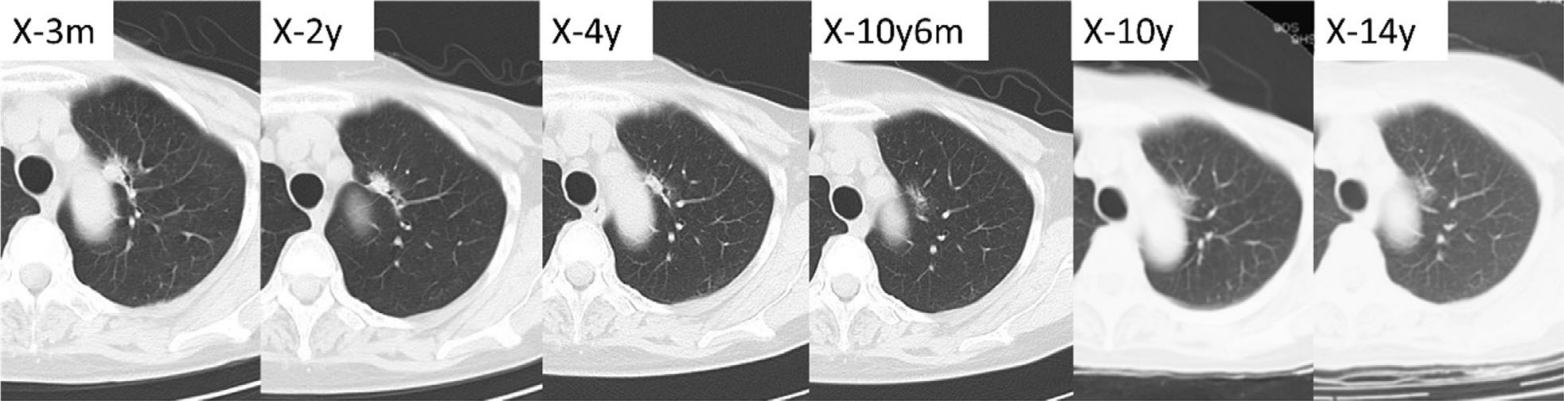
Alterations in chest CT images

A retrospective analysis of the chest CT at the time of breast cancer surgery 14 years prior demonstrated a GGN of less than 20 mm in contact with the aortic arch (Figure 2). The nodule remained unchanged over the next two and a half years. The next year (10 years and a half years ago), the concentration of the central part of the GGN increased; a radiologist attributed it to old inflammation. Although there was a gradual increase in size and an increase in concentration over the next 9 years, these results continued to be attributed to previous inflammation. In the next year (3 months ago), the growth of the tumour was first acknowledged and lung cancer was suspected. She had bone metastases 4 years after the lobectomy, but has survived for five and a half years post‐surgery with treatment with osimertinib.

## DISCUSSION

There are case reports of slowly growing lung cancer, but few cases have been studied for such a long time. Takashima and colleagues showed stepwise progression of replacement‐type lung neoplasms by CT analysis.^1^ They reported lesions that were first known as GGN with a subsequent increase in size, then solid portions appeared in the nodule, and finally, solid portions increased with occasional augmentation of tissue contraction. Min and colleagues described a case in which a pure GGN developed into an invasive lung adenocarcinoma with supraclavicular lymph node metastasis over 125 months.^2^ The solid component of the GGN expanded, and there was a multi‐step development from atypical adenomatous hyperplasia to invasive cancer. Soda and colleagues reported an intriguing GGN case that developed toward invasive adenocarcinoma over 54 months.^3^ The GGN's solid component developed linearly at first, but subsequently, rapidly increased.

For physicians who do not specialize in respirology, reading chest CT is challenging because the intrathoracic organ has different structures that may seal pulmonary nodules. Inevitably, radiologists will be consulted to read the images, but unless they comprehend the nature of the lepidic adenocarcinoma of the lung, they will not be able to point it out correctly. In this case, the CT scans exhibited poor resolution and the presence of GGN was not noted initialy. However, even as the resolution of the CT was enhanced and the solid portion of the GGN became more noticeable, the shadow was diagnosed as old inflammation. However, there was no statistically significant difference when compared to the previous year's CT alone. As a result, it is critical to compare tumour growth over several years.

According to the requirement for pulmonary nodules in lung cancer, low‐dose CT screening and follow‐up observation are suggested by the Japanese Society of CT Screening.^4^ A definite diagnosis is obtained if a pure GGN of 15 mm or greater remains stable or grows on thin CT after 3 months. If it is less than 15 mm, follow‐up is recommended for 2 years; if it increases by 2 mm or more or a solid portion appears, a definitive diagnosis is suggested. According to the guidelines for the management of incidental pulmonary nodules detected on CT images from the Fleischner Society,^5^ if a solid component or growth develops in a GGN >6 mm, resection should be considered. In this case, if the GGN had been found on the initial CT, a definitive diagnosis would have developed. Three and a half years later, because the solid portion of the tumour developed, the definitive diagnosis should have also occurred. According to the Japanese Society of CT Screening, even if the pure GGN remains unaltered for 2 years, annual follow‐up CT is needed for a long period. This case demonstrates the importance of long‐term follow‐up.

This report shows that even a pure GGN can become stage N2 after a long period (14 years). It is important to conduct comparative reading not only in the films of the previous year but also in several years before screening for changes. It is important to educate physicians who do not specialize in respirology about the possibility that GGN with a solid portion may progress into lepidic adenocarcinoma.

## AUTHOR CONTRIBUTIONS

Dr Hiroyuki Miura and Dr Shinichi Goto helped in the conception and design of the work and the acquisition and analysis or interpretation of data for the work. Dr Jun Miura drafted the work and revised it critically for important intellectual content. Dr. Yamamoto diagnosed this cancer pathologically. All authors contributed to the final version of this manuscript and approved it to be published.

## CONFLICT OF INTEREST STATEMENT

None declared.

## ETHICS STATEMENT

The authors declare that appropriate written informed consent was obtained for the publication of this manuscript and accompanying images.

## Data Availability

The data that support the findings of this study are available on request from the corresponding author. The data are not publicly available due to privacy or ethical restrictions.
